# The influence of nursing handover on nurses' mental health: A scoping review

**DOI:** 10.3934/publichealth.2025008

**Published:** 2025-01-15

**Authors:** Margarida A. R. Tomás, Marisa R. Soares, Joaquim M. Oliveira-Lopes, Luís M. M. Sousa, Vânia L. D. Martins

**Affiliations:** 1 Nursing Research, Innovation and Development Centre of Lisbon (CIDNUR), Lisboa, Portugal; 2 Mental Health Nursing Department, Nursing School of Lisbon (ESEL), Lisboa, Portugal; 3 Psychiatric Department, Local Health Unit of Almada-Seixal (ULSAS), Almada, Portugal; 4 Nursing Department, School of Health Atlântica (ESSATLA), Oeiras, Portugal; 5 Comprehensive Health Research Centre (CHRC), Évora, Portugal

**Keywords:** nursing, mental health, patient handoff, review

## Abstract

Nursing handover is essential in clinical practice across various healthcare settings and can significantly impact nurses' mental health. This scoping review aimed to explore and map these implications using the JBI methodology and PRISMA ScR Checklist. It included 11 studies published between 1988 and 2022 from the UK, Australia, USA, South Korea, and Hong Kong, involving over 122 nurses in acute care settings. The findings reveal three major themes: source of psychological discomfort, coping resource, and peer support and cohesion. Negative emotions such as stress, anxiety, dissatisfaction, and tension are linked to handovers, particularly bedside handovers, which raise confidentiality issues and induce scrutiny among nurses. The lack of standardized training and consistent procedures also contributes to stress, especially for newly graduated and less experienced nurses. Conversely, handovers function as structured rituals providing peer support and a sense of control, helping nurses manage psychological demands. To mitigate negative impacts, implementing standardized handover procedures and comprehensive training programs for new nurses is essential. Encouraging open communication and fostering a supportive environment can enhance team cohesion and reduce stress. Future research should measure the impact of different handover practices on nurses' mental health and explore their supportive, social, protective, and restorative functions. This review highlights the critical role of nursing handovers in supporting nurses' mental health and underscores the need for standardized practices to improve the well-being of nursing professionals and the quality of patient care.

## 1. Introduction

Handover, also referred to as shift report or handoff, is a pivotal event in clinical practice across healthcare settings and organizations, often considered a high-risk moment [1]. It involves the transfer of real-time, patient-specific information between healthcare providers, professionals, or caregivers to ensure continuity of care, as well as responsibility and accountability for patients' healthcare and outcomes [2],[3].

The dynamics and complexity of communication processes in healthcare make this a multidimensional and multifactorial process [2] with a myriad of interdependent variables. These variables include personal characteristics of the health professional as well as characteristics of the setting, such as location, procedure, and participants, among others. Even though it can occur at different times, between diverse levels and units of an organization, and even between organizations, handover is the most recurring communicative event across disciplines and specific teams providing patient care (e.g., doctors, nurses, allied health workers) [1]. Nursing handovers are important in healthcare settings during nurses' shift-to-shift rotations [2],[3]. These handovers involve exchanging important patient-related information between nurses and may occur three or more times a day, depending on the shift schedule [4]. It is important to note that nurses are legally responsible and accountable for transferring essential patient-specific information during these handovers [4].

Structurally, fundamental elements suggested in the literature compose the procedures and content of nursing handovers (depicted in Table 1). Standardized communication tools with mnemonic rationales, such as ISBAR/ISBARQ, DeMIST, ISOBAR, ICCCO, SHARED, VITAL, REED, I-PASS, PVITAL, and PSYCH [2],[5],[6], increasingly assure information accuracy and consistency in handover communication through standardization [5].

**Table 1.** Nursing handover elements.

**Handover elements**	**Description**
**Timing**	Occurs during shift changes, patient transfers, admissions, referrals, or discharges.
**Length**	Typically takes between 18 and 50 minutes to complete, depending on the healthcare setting and patient characteristics [6].
**Channel**	Conducted face-to-face, via telephone, through paper forms, or electronic handover platforms.
**Location**	Takes place at the patient's bedside, in a staff room, office, during ward rounds, in hospital corridors [1], or at the reception area of a hospital or clinic.
**Stakeholders**	Involves nurses only; may also involve patients, carers, or relatives; conducted by nurses responsible for handover or head nurses.
**Tools**	Utilizes handwritten forms, computer-generated handover reports, or standardized communication tools based on international guidelines (e.g., ISBAR) [6].

Extensive literature highlights the relationship between handover quality and patient safety [1],[5],[7]. Communication during shift changes is one of the leading causes of sentinel events, and ineffective handovers are also reported as major factors contributing to multiple potential hazards, including the unavailability of required equipment for patients, information omissions, diagnosis errors, treatment errors, disposition errors, and treatment delays [5],[8]. Moreover, the lack of communication between incoming and outgoing nurses entails reduced safety, including delays in treatment, medical errors, and patient injury or death [9].

Hitherto, research has focused on best practices for clinical handover [3],[10], patient involvement, namely bedside handover [10],[11], experiences of clinical handover by nurses, patients, or relatives [3],[12], use of handover tools such as standardized handover forms or electronic handover systems [13],[14], handover improvement interventions [15],[16], and the study of barriers and facilitators [17],[18]. Additionally, the quality of handovers has been considered and reviewed based on content (accuracy and completeness of information), process (including aspects of the transfer environment and behavior of handovers), and results, which encompass everything that occurs after the shift change [19]. Patient safety and satisfaction are the most closely related factors in the literature [19].

Several factors contribute to the quality of handover/handoff communication, which is related not only to the health professional per se but also to organizational and structural issues. Hence, multiple determinants affect the reliability and efficiency of handover communication, as follows: interruptions or distractions due to people (patient, family) solicitation or equipment alarms [6],[18]; workload and insufficient or neophyte nurses, time constraints, poor communication and relations between nurses, and high turnover [8],[20]–[22]; and failure to deliver pertinent information, insufficient handover structure, lack of training in documentation, poor leadership, or safety culture [6],[8].

Acknowledging the relevance of nursing handovers to ensure quality, safety, and continuity of care [5] entails a heavy load on nurses' sense of their caregiving quality. Being aware of the impact of working conditions on nurses' mental health [23],[24] makes it pertinent to wonder if the moment of handovers in nursing clinical practice is, in any way, also related to nurses' mental health. Promoting and protecting nurses' mental health means investing in productivity, adaptability, and resilience toward the difficulties nurses may encounter during their work, which may entail dealing with suffering, uncertainty, and demanding working conditions, among other dire events. Recognizing and reviewing the consequences of nursing handover regarding its mental toll provides insight into its implications for the mental health of nursing professionals and, consequently, the quality of care provided. A preliminary search of PROSPERO, OSF platform, CINAHL Ultimate, MEDLINE Ultimate, the Cochrane Database of Systematic Reviews, and the Joanna Briggs Evidence Synthesis revealed no systematic review protocol or study on this topic. Thus, this scoping review is timely and much needed as it delves into whether nursing handover promotes or hinders nurses' mental health. This review aims to explore and map the implications of nursing handover regarding nurses' mental health. Understanding the pressures and psychological impact associated with nursing handovers provides awareness and contributes to a more thoughtful development of support strategies and specific interventions that promote nurses' mental health.

## 2. Materials and methods

This scoping review acknowledges the paucity of studies that focus on nurses' mental health regarding nursing handover; the search for its clarification entails mapping out the available scientific literature. To that end, the JBI methodology for scoping reviews [25] and PRISMA ScR Checklist [26] guided this review, and the protocol is available at https://osf.io/6584j. Therefore, we used a three-step search strategy to identify published and unpublished primary studies and text and opinion papers. This approach ensured a thorough search and provided a broad understanding of the existing evidence on the topic. First, an initial limited search of MEDLINE Ultimate (via EBSCOhost) and CINAHL Ultimate (via EBSCOhost) allowed the identification of articles on the topic, with the initial terms handover AND nurse* AND “mental health”. Two reviewers (MT and MS) examined the data related to text terms found in titles, abstracts, keywords, and index terms. They identified relevant terms to incorporate into a comprehensive yet targeted search strategy for directing the search process, producing the following search strategy formula: (Nurs* OR Nursing OR Midwife OR Midwives) AND (Handover OR Handoff) AND (“Mental Health” OR Mood OR Anxiety OR Depression OR Stress OR Coping OR Burden OR “Nurses psychology”).

The second stage, accomplished on April 5, 2024, entailed conducting the search strategy formula using four databases: CINAHL Ultimate (via EBSCO), MEDLINE Ultimate (via EBSCO), MedicLatina (via EBSCO), and Scopus. Regarding gray literature, searches included Google Scholar and RCAAP (Repositórios Científicos de Acesso Aberto de Portugal) (for more details see https://osf.io/z5rwc).

The inclusion criteria were based on the Population-Concept-Context framework, but we refined their specifications to ensure they were clear, precise, and had fewer confounding factors. Complying with JBI methodology, we created an a priori protocol that can be revised based on an iterative approach and a better understanding of the literature during the review process. Consequently, we delineated the following parameters described in Table 2.

**Table 2.** Inclusion criteria.

**Inclusion criteria**	**Description**
**Participants**	This scoping review included studies of nurses in clinical practice, regardless of their degree, category, or function. Studies involving nursing students were excluded.
**Concept**	This scoping review focused on studies that explore nurses' mental health, meaning it examined mental health–related concepts (e.g., stress, anxiety, burden) among nurses in clinical practice.
**Context**	This review considered studies focused on shift-to-shift nursing handovers in any healthcare setting. Nursing handovers between departments or healthcare units were excluded.
**Types of sources**	This scoping review analyzed quantitative, qualitative, and mixed-methods study designs, reviews, editorials, and opinions for inclusion. Gray literature such as conference proceedings, theses, dissertations, government documents, policy documents, and books was also considered for inclusion.
**Language**	Sources were restricted to documents in English, Portuguese, or Spanish.
**Time limits**	No time limits were applied to the data; the aim was to provide a comprehensive literature overview.

**Figure 1.**
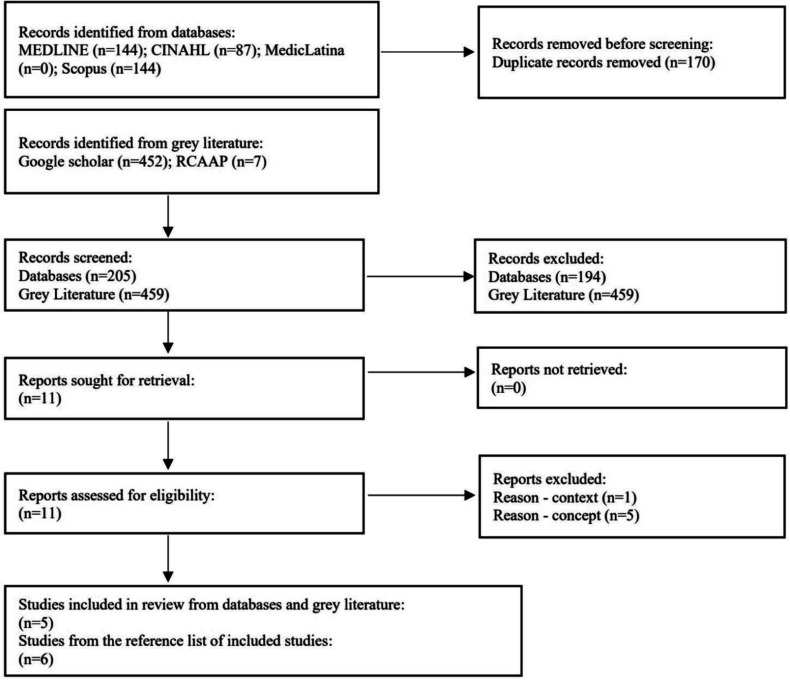
PRISMA-ScR flow diagram.

Study selection proceeded with compiling and uploading all identified studies into Mendeley Reference Manager to identify duplicates and facilitate coordination between reviewers. Following a pilot test involving ten articles (with five articles assigned to MT and VM each), reviewers collaboratively screened titles and abstracts against the inclusion criteria for the review. Potentially relevant papers were moved to a full-text assessment by two independent reviewers (MT and VM) to determine eligibility. Exclusion reasons for full-text papers are documented and available at https://osf.io/bh8g6. In three studies, there was disagreement among the reviewers, which was resolved through discussion and with the involvement of a third reviewer (LS). The study selection and search results are reported in the PRISMA flow diagram (Figure 1) [27].

In the final stage of the search, we examined the reference lists of included documents to identify additional relevant sources. Three articles presented criteria for possible inclusion, and the authors were contacted via e-mail through ResearchGate, although none replied.

Data extraction from the included studies followed a standardized extraction form. This form included the title, author, year of publication, country of origin, type of study, participants, healthcare setting, study aims, mental health concepts, and key findings. During data extraction, we added a category of data extraction, namely the type of handover, as we needed to point out the procedural configuration of the study's handover. Two reviewers (MT and VM) previously tested the extraction form, independently extracting information from five records and assessing their congruency. MT completed the data extraction, and VM verified the correctness. This rigorous process ensures the accuracy and reliability of the data presented in this review (full data extraction available at https://osf.io/qzh9f).

All team members conducted data analysis based on the study's objectives and corresponding review questions, providing insights into the major themes that emerged from the data. We used qualitative content analysis to synthesize the mapping data and identify themes effectively. We also included relevant tables to support our findings.

## 3. Results

This scoping review includes 11 studies published between 1988 and 2022. The studies originate from the United Kingdom (*n* = 4), Australia (*n* = 4), United States of America (*n* = 1), South Korea (*n* = 1), and Hong Kong (*n* = 1) [28]–[38]. Most of the included studies employ a qualitative methodology (*n* = 9), comprising four ethnographic studies, two phenomenological studies, one qualitative case study, one qualitative descriptive study, and one qualitative exploratory study. Additionally, two theoretical papers are incorporated. Regarding the healthcare setting, all studies take place in (or refer to) hospital environments, namely acute care settings, predominantly medical and surgical wards, and intensive care units (Table 3). These studies collectively involved a wide range of nursing professionals, from newly graduated nurses to senior nursing directors, including more than 122 nurses (considering that six studies do not enumerate the number of study participants).

Overall, nurses typically conduct verbal shift reports, mainly face-to-face, in a designated staff area, such as an office, where outgoing nurses report their patients to the incoming nurses, even though the nurses in charge of handovers differed with the hospital system (*n* = 9). Two studies presented a handover report that starts with a “global handover” performed by the head nurse or the nurse in charge of the shift to provide an overview of all patients, followed by a bedside handover between the outgoing–incoming nurses [28],[29]. Kim and colleagues found that in primary nursing, such as in intensive care units, the nurse directly responsible for a patient would hand them over to the next nurse in charge. In team nursing, the handover responsibilities were with the nurse in charge or the senior nurse, and the other team nurses would listen to the handover instructions together [30]. This process also involved using electronic medical records (EMRs) and Cardex, which could be non-contact (proceedings not elucidated by authors) or face-to-face [30]. Five studies mentioned bedside handovers, where the patient and sometimes their family or caregivers are present [28],[29],[31]–[33]. This type of handover focuses on the report of a specific patient between the nurse in charge of their care and the nurse who will be taking over in the patient's unit (at his bedside). Therefore, the significant distinction between the type of handover present in the included studies relates to the location (office or bedside) and the nurses providing the handover (if the nurse in charge of handover or nurse in charge of the patient).

**Table 3.** Summary of included studies.

**Author (year)**	**Country**	**Type of study**	**Healthcare setting**
**Strange (1996)** [28]	United Kingdom	Ethnographic study	Hospital ward (for intensive treatment)
**Manias & Street (2000)** [29]	Australia	Critical ethnography study	Critical care unit of a public teaching hospital
**Kim et al. (2022)** [30]	South Korea	Qualitative descriptive study	Wards and intensive care units of small and medium-sized hospitals
**Happell et al. (2013)** [31]	Australia	Qualitative exploratory study	Acute care hospital (pediatric, surgical, and medical wards)
**Hopkinson (2002)** [32]	United Kingdom	Phenomenological study	Acute hospital medical wards
**Thurgood (1995)** [33]	United Kingdom	Theoretical analysis	Not specified
**Chung et al. (2021)** [34]	Hong Kong (China)	Descriptive phenomenological study	Public acute care hospitals
**Evans et al. (2008)** [35]	Australia	Qualitative case study	Medical ward of metropolitan teaching hospital
**Wiltshire & Parker (1996)** [36]	Australia	Theoretical analysis	General hospital settings
**Wolf (1988)** [37]	United States of America	Ethnographic study	Medical unit of a large urban hospital
**Lally (1999)** [38]	United Kingdom	Ethnographic observational study	Surgical ward in general hospital

Understanding mental health involves the intricate interplay of various factors within a dynamic and holistic framework, which includes constructs such as anxiety, stress, or coping. Consequently, we identified three themes that focus on how nurses' mental health is affected by handovers: (1) source of psychological discomfort; (2) coping resource; and (3) peer support and cohesion (see Table 4). The following sections describe each theme.

### 3.1. Source of psychological discomfort

Handovers can constitute a source of negative feelings, including fatigue, dissatisfaction, burden, tension, uneasiness between nurses, criticism, bullying, frustration, shame, insomnia, lack of confidence, dissatisfaction, fear, and anxiety. Bedside handovers have been found to raise patient confidentiality issues [33], causing stress for nurses who feel uncomfortable discussing sensitive information in front of patients and their relatives [31]. Additionally, tension arises when nurses must discreetly communicate sensitive information, particularly when caring for dying patients, further adding to their stress [32]. In particular, Manias and Street's critical care unit study revealed another source of psychological distress regarding bedside handovers, which entails a sense of scrutiny from the incoming nurses, which induces fear and anxiety in the outgoing nurses [29]. They felt they were being examined or critiqued when questions or requests for more patient information occurred, which usually focused on gaps or faults rather than achievements or deeds. Correspondently, the “tyranny of tidiness” constituted a pervasive feature during bedside handover, as the outgoing nurse felt she had to hand off the patient with an expected standard determined by nurses A or B, and failure to reach those expectations resulted in feelings of guilt, which meant that the outgoing nurse “did not make the bed area tidy by the time of the bedside handover” [30]. Simultaneously, “the tyranny of business” meant that the incoming nurses pointed out uncompleted or subpar activities of the outgoing nurse, failing to acknowledge the difficulty and high demand shift the outgoing nurse endured, and even presenting “unappreciative comments” [30]. Moreover, some nurses utilized guilt as a form of punishment to assert their power.

**Table 4.** Type of handover and thematic findings of included studies.

**Author (year)**	**Type of handover**	**Thematic findings**
**Strange (1996)** [28]	Clinical handover by the nurse in charge of a shift (face-to-face in a designated staff room). Bedside handovers were also performed, but the study did not investigate them.	Coping resource Peer support and cohesion
**Manias & Street (2000)** [29]	Clinical handovers (face-to-face in a designated staff room) designated as “global handover” and followed by bedside handovers.	Source of psychological discomfort
**Kim et al. (2022)** [30]	Clinical handovers (face-to-face in a designated staff room) and non-contact handovers, with the aid of electronic medical records (EMRs) and Cardex in all hospitals. The nurses in charge of handovers differed with the hospital system.	Source of psychological discomfort
**Happell et al. (2013)** [31]	Bedside handovers	Source of psychological discomfort
**Hopkinson (2002)** [32]	Six wards have clinical handovers (face-to-face in a designated staff room); two wards have bedside handovers.	Source of psychological discomfort Coping resource
**Thurgood (1995)** [33]	“Verbal handover reports” (office and bedside, but no clear distinction)	Coping resource Peer support and cohesion
**Chung et al. (2021)** [34]	Clinical handovers (face-to-face in a designated staff room)	Source of psychological discomfort
**Evans et al. (2008)** [35]	Clinical handovers (face-to-face in a designated staff room)	Coping resource Peer support and cohesion
**Wiltshire & Parker (1996)** [36]	Clinical handovers (face-to-face in a designated staff room)	Coping resource Peer support and cohesion
**Wolf (1988)** [37]	Clinical handovers (face-to-face in a designated staff room)	Coping resource Peer support and cohesion
**Lally (1999)** [38]	Clinical handovers (face-to-face in a designated staff room)	Peer support and cohesion

Manias and Street referred to “the global handover”, which happened in a private staff room before the bedside handover [29]. Nurse coordinators mainly performed it to help them understand patients' overall status in the ward. The current communication process inhibited clinical nurses from participating or asking for more patient information, causing frustration among nurses who felt left out, even though the handover process was seen as necessary. As a result, the global handover process seems to have ritualistic characteristics that give nurse coordinators a sense of control and authority in the work environment [29].

Two other studies pointed out difficulties that neophyte nurses may experience during handover, albeit viewing clinical handover with reverence and as an essential nursing practice that ensures continuity of care [30],[34]. Kim and colleagues mentioned that small and medium-sized hospital wards lack standardized handover training [30]. This leads to inconsistency and stress among nurses, prevailing conformity to the handover style dictated by the head or senior nurses of a respective ward. Newly hired nurses often receive inadequate training, leading to adaptation difficulties and anxiety [30]. Some experience bullying during handovers, which are felt as a moment for inspection, attributing blame and criticism toward the new nurse's performance, which is considered subpar, and creating an uncomfortable atmosphere among the nursing team [30]. In turn, senior nurses found handovers burdensome due to the time spent on training new nurses. Face-to-face verbal handovers assisted by electronic medical records were common, but non-contact handovers were also mentioned [30]. While senior nurses preferred non-contact handovers, most nurses preferred face-to-face handovers for better concentration and understanding [30]. Nevertheless, inefficient handovers due to inexperience or duplication of information led to fatigue and dissatisfaction [30].

Chung and colleagues also stated that new general nurses often experience significant stress and anxiety during clinical handovers, particularly when handovers are perceived as chaotic or inefficient, leading to feelings of frustration and shame and sometimes even affecting their quality of life, such as causing insomnia [34]. These negative feelings are exacerbated when handing over to senior nurses, who may question their reports more critically and express dissatisfaction with their performance. Feeling unprepared for the handover due to highly demanding work during the shift, which did not allow the preparation of the handover reports, also contributed to anxiety and lack of confidence [34]. Additionally, most participants explained that a significant difficulty in conducting clinical handovers was due to unsystematic reporting, making it difficult to present the patient's current status logically [34]. Conversely, some participants reported receiving support and close supervision from their senior colleagues, which allowed them not to experience too much anxiety or stress toward clinical handover practice [34].

### 3.2. Coping resource

Nursing handovers serve as a vital coping resource for nurses, helping to manage clinical practice's intrinsic psychological and emotional demands. As a critical transition space embedded in a ritualistic framework, it functions to alleviate anxiety among nurses by establishing control and predictability over a vulnerable period of transition of power, control, and responsibility for patient care [28],[35],[36]. These rituals create a structured, tacitly regulated environment that allows outgoing nurses to debrief and manage their emotional disturbances. In contrast, incoming nurses prepare cognitively and emotionally for their shift [36], and organize and prioritize their nursing care, reducing uncertainty and helplessness, particularly in dynamic ward environments where patient status can change rapidly [28].

Depicted as an occupational ritual of socialization [37], handover enables nurses to process distressing experiences in a supportive, non-judgmental environment, facilitating the collective psychological management of difficult experiences [36]. This ritual allows nurses to voice complaints, share humor and concerns, deal with body decay, death, and other anxiety-inducing situations, and collaboratively resolve shared patient and professional problems [36],[37]. Hence, for incoming nurses, it provides the necessary preparation for the shift before them, providing more than patient information, allowing them to process the expressed abjection of the outgoing nurses and to process them through the rationalist and collective dimensions of the handover [36]. The categorization of patients during handovers, often through stereotyping, serves as a psychological defense mechanism, helping nurses cope with the complexities of their roles [35]. Hopkinson emphasized the supportive nature of handovers as an unexpected finding of his study, which sought to understand how nurses can be supported in caring for dying people [32]. Handovers helped some nurses alleviate anxiety, stress, and frustration, facilitating discussions about their experiences and providing essential information that aids their emotional preparation for the upcoming shift [32].

The ability to share responsibilities, commitment to patient care, and associated anxieties with peers provide significant emotional support and control, reinforcing this ritual's importance in managing the emotional demands of nursing practice [32],[37]. Thus, a positive and inclusive handover environment diminishes the negative impacts of staff hierarchy [33]. It provides informal feedback from peers and formal appraisals from managers, which help nurses reduce stress and anxiety and identify their strengths and weaknesses [33].

### 3.3. Peer support and cohesion

Nursing handovers provide a structured environment that fosters emotional ties, group identity, teamwork, and shared responsibility among nurses, thus promoting peer support and cohesion.

In clinical nursing, handovers are ritualistic events that foster group cohesion, especially during informal social gatherings before the formal handover process. These gatherings involve casual conversations and shared practices, which help develop trust and a sense of teamwork among incoming nurses and create a supportive atmosphere in which nurses can emotionally connect with their colleagues [28],[35]. As a result, group identity and solidarity emerge, enabling the management of underlying workplace anxiety, difficult experiences, and professional problems [35]–[37].

Thurgood pointed out that effective handover reports are significant for fostering teamwork [33]. Nurses involved in these reports experience increased morale, motivation, and cooperation [33]. This positive environment encourages information sharing and supports the growth of team morale, which is essential for maintaining a cohesive nursing team [33]. The collaborative nature of completing unfinished tasks post–shift reports symbolizes nurses' continuous commitment and shared responsibility [37]. This ritual reinforces the transfer of patient ownership and ensures the transmission of clinical knowledge, standards of care, and values from shift to shift. Notwithstanding, handovers serve a social function by managing and identifying “deviant cases”, where non-verbal comments and unwritten rules guide social behavior and reinforce group norms [28]. For example, after the handover, the oncoming shift members proceed to the ward as it is considered impolite to keep the other shift waiting [28]. Additionally, there is an unwritten rule that the handover report should not be too long. There was a nurse who had a reputation for long reports, and it became a standing joke to ask her if she knew the Apgar score of patients who were quite elderly [28].

Lally highlighted that the inter-shift handover process, involving all incoming and outgoing nurses in a staff room, is more than just transferring patient care information; it is a ritual that ensures continuity of care and serves as a forum for team building [38]. During these handovers, nurses communicate shared goals and values related to nursing practice, enhancing a communal value system [38]. This process is vital for promoting team unity and maintaining high care standards, demonstrating that handovers are essential for team cohesion rather than just traditional or routine forms of communication. The ritualistic nature of handovers serves a deeper purpose beyond the transfer of information; it fosters a sense of community and support among nursing staff [38]. Prioritizing a positivistic stance on technical, rational, and scientific validation within the nursing profession might unintentionally lead to undervaluing the handover as a source of peer support and cohesion [28],[38].

## 4. Discussion

The findings of this scoping review highlight the significant impact of nursing handovers on nurses' mental health. These have substantial implications for clinical practice, reaching far beyond the patient-specific information delivered between nurses to ensure the continuity and safety of patient care.

Nursing handovers can be a significant source of psychological discomfort, manifesting in various negative emotions such as fatigue, dissatisfaction, burden, tension, uneasiness, criticism, bullying, frustration, shame, insomnia, lack of confidence, fear, and anxiety. Thurgood [33] and Happell et al. [31] found that bedside handovers could raise patient confidentiality issues, causing stress for nurses who feel uncomfortable discussing sensitive information in front of patients and their families. This tension is exacerbated when nurses must discreetly communicate sensitive information, particularly in caring for dying patients [32]. However, nurses recognize that bedside handovers help build rapport with patients and families, as highlighted by Hopkinson [32]. Notwithstanding, bedside handovers have become increasingly popular in recent years due to improved patient and nurse satisfaction and reduced miscommunication, errors, and costs [10]. Thus, patients support bedside handover because it empowers them to be involved in their nursing care and enables them to participate in care decisions [39]. As a result, best practice recommendations establish that bedside clinical handover should be standardized, patient-centered, and personalized to ensure confidentiality and respect individual preferences for involvement [10].

Manias and Street [29] identified additional sources of psychological distress within the intensive care unit (ICU). They noted that during bedside handovers, outgoing nurses often felt heightened scrutiny and anxiety, as incoming nurses often focused on faults and incomplete tasks rather than acknowledging achievements. This emphasis on perfection and productivity led to guilt and stress among the outgoing nurses. Ahn and colleagues [9] also corroborated these findings, describing how ICU nurses expressed feeling belittled by criticism from incoming nurses and were held to an unrealistic expectation of perfection, which in turn made outgoing nurses feel guilty and compelled to apologize for any missed or incomplete tasks, regardless of the demanding nature of their shift. Indeed, Chung and colleagues [34] reported that new nurses working in sub-acute settings with stable patients considered preparing reports with ample time, with some receiving full support and close supervision from senior colleagues, reducing anxiety and stress.

The global handover, performed in a private staff room by nurse coordinators, excluding clinical nurses from participating [29], entails recognizing the need for partnership embedded in mutual respect and cooperative relationships [9]. In order to enhance communication, it is essential for the nurse receiving information to engage actively during the handover process, taking into account any potential differences in understanding that may occur during transitions of care [40]. The absence of chances to confirm information and the fear of being seen as incompetent are barriers to obtaining information and produce ineffective communication [9].

Newly graduated and less experienced nurses also face significant stress and anxiety during handovers, even described as bullying, partly due to a lack of handover training and standardized procedures and due to a lack of efficiency in performing nursing tasks and shift reports [30],[34]. Best practice recommendations establish a standardized, structured approach for inter-shift clinical handover, recognizing that different settings may require unique system adaptations, as a one-size-fits-all approach may not be appropriate [41],[42]. Even so, employing an information checklist during patient handover is advised to enhance the handover process and care delivery, which may benefit from integrating electronic systems to support the process [41],[42].

Standardizing handover procedures across healthcare settings means reducing variability and ensuring all nurses, especially newly graduated ones, receive adequate training that must be secured through proper integration training [30],[34]. Establishing a consistent and time-sensitive preceptorship program, supervised by senior and experienced nursing colleagues, supports newly graduated nurses in adapting to their new working environment [34].

The ritualist nature of nursing handovers becomes evident and was explored in five studies [28],[29],[35]–[37], constituting the basis for establishing the handover as a coping resource for nurses. As a ritual, it establishes a set of collective tacit conducts that serve to create a structured and predictable routine during a vulnerable transition that marks the start and the end of a shift; it enables nurses to manage anxiety and other emotional disturbances and to share values, responsibilities, and hardships [28],[35]–[37]. Moreover, nurses can feel abjection as a human reaction to what they perceive as fundamentally disturbing or vile [36]. This can happen when there is a breach of bodily boundaries, like exposure to bodily fluids or severe wounds, which can feel like an intrusion of the unclean or impure into their sense of cleanliness and integrity. Managing this feeling of abjection is an essential aspect of nursing handover [36]. Stereotyped comments, a lay moral–based discourse, are considered a defense mechanism that allows nurses to present incoming nurses with new patients, or even patients' status, as less unfamiliar [35]. These are associated more with how the nurses feel toward the patient than the appraisal of the patient itself [35]. The negative or positive stereotyping of specific patients was subtly conveyed and formed an unconscious bias reflected in the handover practice, concealing the nurses' true feelings of affection or aversion toward their patients and masking the emotional impact of nursing on nurses' feelings [35]. Regulating anxiety and other distressing experiences through handovers means having the opportunity to debrief and manage emotional disturbances of the outgoing shift among peers—recognized as genuinely able to comprehend the feelings and work pressure—and to prepare cognitively and emotionally for the incoming shift, reducing uncertainty and helplessness [32],[35]–[37].

Therefore, nurses must consider the protective, supportive, and restorative functions of handovers. This is particularly relevant when handovers occur in a designated staff room, offering a private setting for nurses to express emotions and discuss experiences without the presence of patients or families, unlike bedside handovers [28],[32],[36].

One other aspect relevant to nurses' mental health relates to fostering peer support and cohesion among nurses, which highlights the social function of the handover [28]. Handover rituals are important for forming emotional ties among nurses, which are essential for group cohesion, fostering a sense of collective identity among nursing staff, and promoting peer support and teamwork [33],[35]–[37]. Lally [38] highlighted that the handover process is more than just transferring patient care information; it is a ritual that ensures continuity of care and is a forum for team building. During handovers, nurses communicate shared goals and values related to nursing practice, enhancing a communal value system [28],[37],[38]. This process is vital for promoting team unity, reducing hostility between the nursing team, and maintaining high care standards, demonstrating that handovers are essential for team cohesion rather than outdated communication methods [35],[38].

The implicit functions of nursing handovers, as recognized by these findings, are often hidden [32],[35]. Even though nurses see handover as necessary for continuity of care, information transfer, and student teaching, many nurses do not perceive the handover as a ritual [28]. Critics of the ritualistic nature of nursing work consider rituals as repetitive, outdated, purposeless, and void of evidence-based practice [28],[37]. However, ritualized behavior emerges as a way for nurses to alleviate anxiety and establish control, namely in demanding settings such as acute care hospital services [28],[35],[37]. Additionally, attending handover for nurses seems to be a cultural rule, ensuring social order by enforcing cohesion interaction, upholding common nursing values, and maintaining exclusivity within their group [43].

Current research extends a greater focus on explicit functions related to communicating information from one nurse/shift to the next and handing over responsibility for the patient [35]. Two types of projects were highlighted in a recent systematic review of quality improvement projects aimed at enhancing the inter-shift nursing handover process [15]. These are the implementation of standardized communication tools and patient-participation bedside handover [15]. The outcomes measured included patient safety, handover time, patient and caregiver participation, and nurse perception and satisfaction [15]. The quality improvement projects that were reviewed do not emphasize the supportive, social, protective, or restorative aspects of handover, which are acknowledged to play a significant role in the mental well-being of nurses. Instead, these projects mainly assess outcomes related to patient safety and efficiency.

### 4.1. Recommendations

To enhance nursing handover practices and support nurses' mental health, it is fundamental to recognize the psychological and social functions of handovers, embedded in a ritualistic nature. This provides a sense of control and predictability as well as regular, structured interactions among peers, which are essential for emotional support and coping with the high-stress nature of nursing work.

Moreover, standardizing handover procedures by implementing structured protocols, such as the SBAR framework, can help alleviate the psychological burden on nurses by providing a predictable and structured process, particularly in neophyte nurses. Comprehensive training programs for newly graduated and less experienced nurses, properly supervised by senior nursing colleagues, help newly graduated nurses adapt to their new working environment more smoothly, reducing stress and anxiety [30],[34].

Encouraging open and transparent communication during handovers is vital for creating a supportive environment. Nurses should feel comfortable expressing concerns and sharing experiences without fear of criticism. This openness fosters mutual support and collaboration, enhancing team cohesion and morale [32],[37].

Bedside handovers, despite potential confidentiality issues, should be conducted in a manner that respects patient privacy and involves them in their care. Standardizing patient-centered bedside handovers improves patient and nurse satisfaction and reduces miscommunication [10],[31].

From a policy perspective, healthcare institutions should develop comprehensive handover policies that include standardized procedures and training guidelines. These policies should emphasize the importance of confidentiality and address power dynamics within nursing teams, such as hierarchical relationships where senior nurses may dominate or intimidate less experienced nurses, to prevent handovers from becoming sources of bullying or criticism. Promoting a culture of mutual respect and collaboration is key to maintaining a supportive work environment [8],[29],[30].

Future research should focus on quantitative studies, to systematically measure the impact of different handover practices on nurses' mental health outcomes, and also qualitative studies, which focus on nurses' experience of handover and associated emotional and psychological challenges. Expanding research to include a variety of healthcare settings beyond hospitals is essential for a comprehensive understanding of handover practices. Further research should also explore the supportive, social, protective, and restorative functions of handovers, which play a critical role in nurses' mental health [28],[30],[34],[37].

### 4.2. Limitations

The primary limitation of this review is its reliance on studies conducted mainly in hospital settings, which may not fully capture the experiences of nurses with handovers in other healthcare environments. Additionally, most studies are qualitative, and no critical appraisal of the quality of the included studies was conducted, which limits the reliability and generalizability of the findings. Furthermore, we were unable to access three studies despite efforts to contact the authors, necessitating their exclusion.

Despite these limitations, this review provides valuable insights into the critical role of nursing handovers in supporting nurses' mental health.

## 5. Conclusions

This scoping review underscores the complex interplay between handover practices and nurses' mental health. The findings indicate that handovers can be both a source of psychological discomfort and a vital coping resource, highlighting the need for standardized and supportive handover practices across healthcare settings. The ritualistic nature of handovers provides structure and predictability, which are essential for managing the psychological and emotional demands of nursing practice. This structure helps nurses prepare for their shifts, manage anxiety, debrief from emotionally challenging experiences, and foster a supportive environment that enhances team cohesion and peer support. Nevertheless, it is recognized that the location of the handover—bedside or private office—the involvement of new nurses to the team, and the demands inherent to particular settings, such as dying patients or critical units, entail different benefits and challenges to nurses' mental health. Further research must consider the explicit functions of handover—transfer of information and patient responsibility—as well as the implicit functions that concern supportive, social, protective, and restorative purposes. Understanding these can help design better handover protocols that support nurses' mental health while maintaining high standards of patient care.

## Use of AI tools declaration

The authors declare they have not used Artificial Intelligence (AI) tools in the creation of this article.
